# Deterioration of the Gαo Vomeronasal Pathway in Sexually Dimorphic Mammals

**DOI:** 10.1371/journal.pone.0026436

**Published:** 2011-10-19

**Authors:** Rodrigo Suárez, Pedro Fernández-Aburto, Paul R. Manger, Jorge Mpodozis

**Affiliations:** 1 Laboratorio de Neurobiología y Biología del Conocer, Departamento de Biología, Facultad de Ciencias, Universidad de Chile, Santiago, Chile; 2 School of Anatomical Sciences, Faculty of Health Sciences, University of the Witwatersrand, Johannesburg, Republic of South Africa; VIB & Katholieke Universiteit Leuven, Belgium

## Abstract

In mammals, social and sexual behaviours are largely mediated by the vomeronasal system (VNS). The accessory olfactory bulb (AOB) is the first synaptic locus of the VNS and ranges from very large in Caviomorph rodents, small in carnivores and ungulates, to its complete absence in apes, elephants, most bats and aquatic species. Two pathways have been described in the VNS of mammals. In mice, vomeronasal neurons expressing Gαi2 protein project to the rostral portion of the AOB and respond mostly to small volatile molecules, whereas neurons expressing Gαo project to the caudal AOB and respond mostly to large non-volatile molecules. However, the Gαo-expressing pathway is absent in several species (horses, dogs, musk shrews, goats and marmosets) but no hypotheses have been proposed to date to explain the loss of that pathway. We noted that the species that lost the Gαo pathway belong to Laurasiatheria and Primates lineages, both clades with ubiquitous sexual dimorphisms across species. To assess whether similar events of Gαo pathway loss could have occurred convergently in dimorphic species we studied G-protein expression in the AOB of two species that independently evolved sexually dimorphic traits: the California ground squirrel *Spermophilus beecheyi* (Rodentia; Sciurognathi) and the cape hyrax *Procavia capensis* (Afrotheria; Hyracoidea). We found that both species show uniform expression of Gαi2-protein throughout AOB glomeruli, while Gαo expression is restricted to main olfactory glomeruli only. Our results suggest that the degeneration of the Gαo-expressing vomeronasal pathway has occurred independently at least four times in Eutheria, possibly related to the emergence of sexual dimorphisms and the ability of detecting the gender of conspecifics at distance.

## Introduction

The mammalian vomeronasal system (VNS) mediates in the perception of pheromones and the orchestration of bodily responses related to social and sexual interactions [1]. The accessory olfactory bulb (AOB) is the first synaptic locus of the VNS and receives afferents from sensory neurons of the vomeronasal organ (VNO). The relative size of the AOB ranges from very large in South American caviomorph rodents [2], [3], to small in ungulates, carnivores and monkeys [4], [5], to its complete absence in Old World monkeys and apes, all flying foxes and most micro bats, elephants and sea cows, all cetaceans, and some seals [6], [7], [8], [9].

Two anatomical and functionally distinct pathways have been described in the mammalian VNS [2], [3], [10], [11], [12], [13], [14]. Neurons located near the apex of the VNO lumen express pheromone receptors of the V1R family, which are coupled to Gαi2-protein, and send projections to glomeruli located in the rostral half of the AOB. On the other hand, neurons at the base of the VNO express V2R receptors coupled to Gαo-protein and send projections to glomeruli of the caudal AOB [10], [12], [15], [16], [17], [18], [19], [20]. Each pathway has a distinct sensorial specificity: while V1R receptors have a small extracellular ligand-binding N-domain [17], [18] and show high affinity for small and volatile molecules [21], [22], V2R receptors have a large N-domain [17], [18], [19] and show high affinity for large non-volatile molecules, such as urinary proteins and exocrine gland-secreted peptides [23], [24], [25], [26].

Although it was initially thought that both pathways were present in all mammals with a functional VNS [27], subsequent studies showed that the Gαo-positive pathway was absent in goats [4], shrews, horses, dogs and marmosets [5]. In these species the vomeronasal nerve and glomeruli express Gαi2-protein only, uniformly throughout the AOB. Accordingly, later genomic studies showed that in dogs, cows, macaques, chimpanzees, and humans, the complete V2R gene family underwent pseudogenisation, i.e., loss of function by accumulation of mutations, [28], [29], further suggesting that the loss of the V2R-Gαo pathway has occurred at least twice independently; in the lineages leading to the superorder Laurasiatheria and to the order Primates (superorder Euarchontoglires). However, to the best of our knowledge, no ecological context has been proposed to relate with these events of sensory loss.

**Figure 1:**
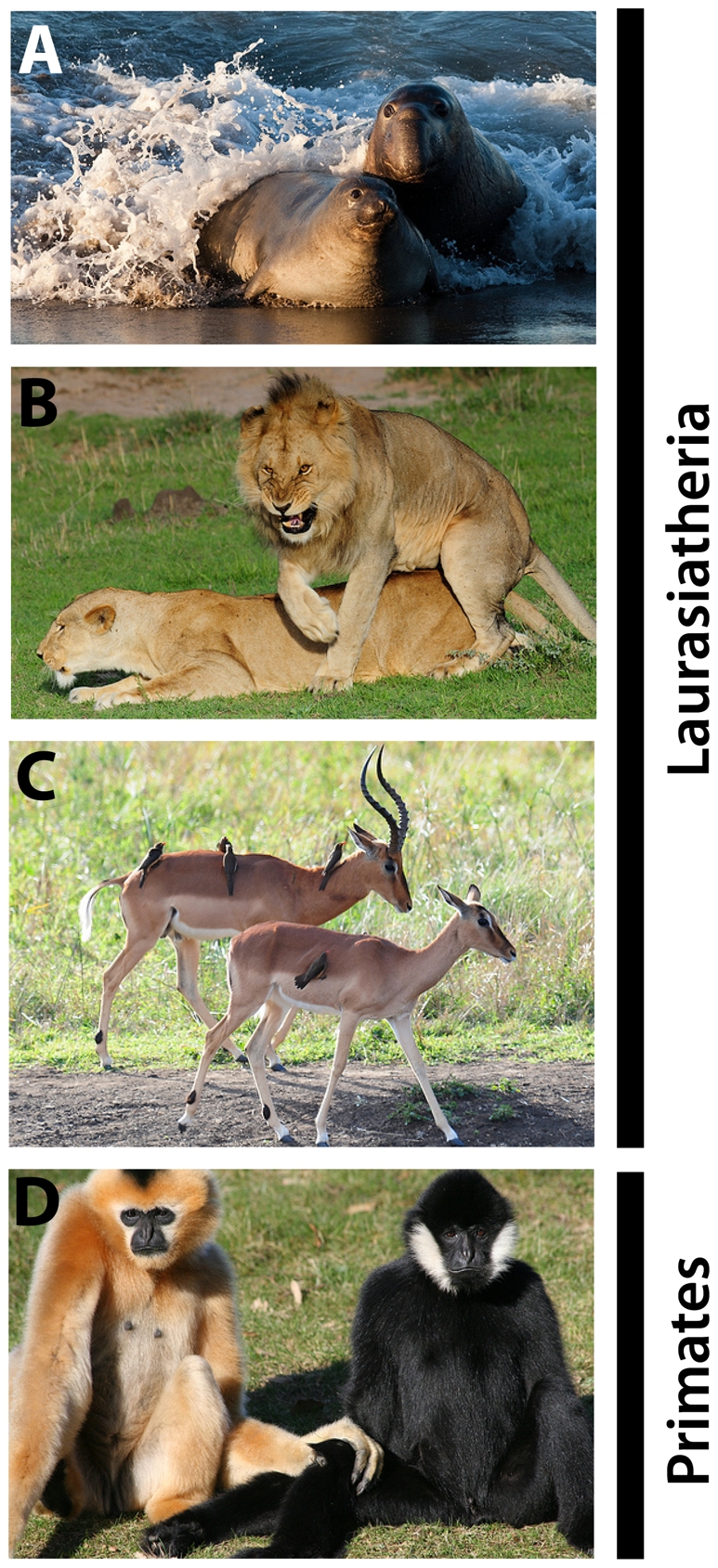
Sexual dimorphisms in Laurasiatheria and Primates. Males and females differ in body size and/or shape (A, elephant seal *Mirounga leonina*, Phocidae; B, lion *Panthera leo*, Felidae), presence of accessories such as horns or tusks (C, Impala *Aepyceros melampus*, Bovidae) and/or fur pattern/colouration (D, Gibbon *Nomascus leucogenys*, Hylobatidae). Pictures by Mike Baird (A), Vince Smith (B,C), and Linda Brosens (D) under a creative commons license.

We noticed that practically all species of Primates and Laurasiatheres show visually conspicuous sexual dimorphisms, expressed not only in body size and shape but also in secondary traits such as hair/fur colouration patterns, the presence of accessories like horns or tusks, and behavioural displays (Fig. 1) [30], [31]. This observation prompted us to ask whether and to which extent the association between the absence of the Gαo-expressing pathway and the presence of sexual dimorphisms can be generalised among mammals.

Sexual dimorphisms have also evolved in Afrotheria, a basal superorder that includes elephants, tenrecs and hyraxes [30], [32], [33], [34], [35], and in some Old World rodents, such as the squirrel-related clade (suborder Sciuromorpha) [36], [37]. Thus, to assess whether similar events of deterioration of the Gαo-expressing vomeronasal pathway may have occurred in dimorphic species of these clades, we studied G-protein expression in the AOB of the cape hyrax (*Procavia capensis*, Afrotheria; Hyracoidea) and the California ground squirrel (*Spermophilus beecheyi*, Rodentia; Sciuridae). Interestingly, we found that both species have also lost the Gαo-expressing vomeronasal pathway.

## Results

We studied Gαi2 and Gαo expression in AOB glomeruli of the California ground squirrel *Spermophilus beecheyi* and the cape hyrax *Procavia capensis*.

**Figure 2:**
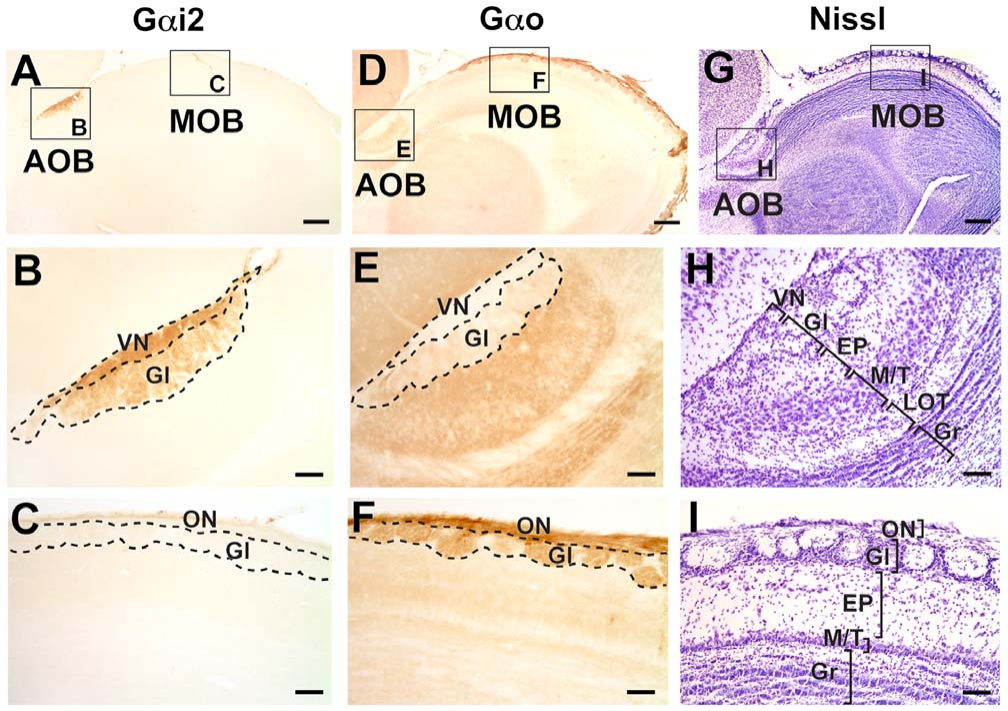
Patterns of Gαi2 and Gαo expression in the accessory olfactory bulb (AOB) of the ground squirrel *Spermophilus beecheyi*. Sagittal sections through the olfactory bulb reveal that the AOB is located dorsocaudal to the main olfactory bulb (MOB) and expresses Gαi2 throughout its rostrocaudal extent (A, B), at the vomeronasal nerve layer (VN) and glomerular layer (Gl) but not in glomeruli of the MOB (C). Gαo expression is restricted to MOB glomeruli (D, F) and deep layers of the AOB, including some expression at the Gl, but not at the VN, layers (E). Panels G, H, and I correspond to cresyl violet stained sections, where cell layering and relative sizes of AOB and MOB can be appreciated. EP, external plexiform layer; Gr, granule cell layer; LOT, lateral olfactory tract; M/T, mitral/tufted cell layer. Dorsal is to the top and anterior is to the right. Scale bar: 500 µm in A, D and G; 200 µm in B, C, E, F, H and I.

The AOB of *S. beecheyi* is located at the dorsocaudal extent of the main olfactory bulb (MOB). It is very small in relation to the MOB, specially when compared with Caviomorph (South American) rodents [2], [3] (Figure 2A, 2D and 2G).

*S. beecheyi* AOB exhibit the characteristic six-layered cytoarchitecture common to all rodents (Figure 2H). All layers (VN, vomeronasal nerve; Gl, glomerular; EP, external plexiform, M/T, mitral and tufted, LOT, lateral olfactory tract, and Gr, granular layer) appear clearly defined with Nissl stain and Gαo immunolabeling: (Figure 2E and 2H). Interestingly, the M/T layer is distributed in a wide area up to 7–9 cells in depth, contrasting with the narrow width that this layer has in all other rodents described so far.

The pattern of expression of Gαi2 protein in *S. beecheyi* AOB is depicted in Figure 2A–C. There is an intense labelling in the VN and Gl layers (Figure 2B), spanning the entire AOB. We observed no expression of Gαi2 in MOB glomeruli (Figure 2C). Surprisingly, the pattern of expression of Gαo protein at the AOB and MOB resembles the situation described in Laurasiatheres and Primates [5], namely, while densely expressed in the olfactory nerve (ON) and glomerular (Gl) layers of the MOB (Figure 2F) and to a lesser extent in the deep layers of the AOB (Figure 1E), the VN layer, which exclusively contains vomeronasal axons, is devoid of Gαo-expression (Figure 2E). Note that Gαo expression is restricted to the parenchyma between AOB neuronal bodies and is almost absent at the LOT (Figure 2E). The clear stratification of MOB layers (Figure 2I) is resembles that of all other mammals studied to date, thus suggesting a highly conserved organisation.

**Figure 3:**
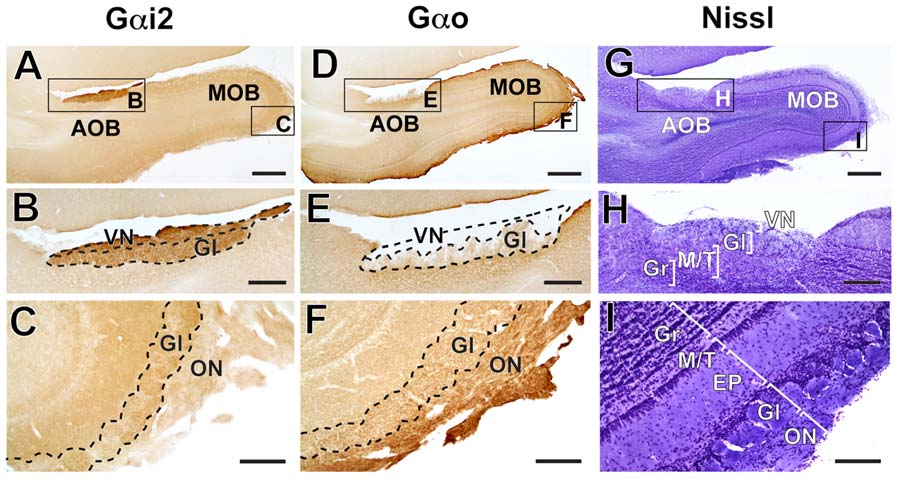
The accessory olfactory bulb (AOB) of the cape hyrax expresses Gαi2 but not Gαo. Similar to *S. beecheyi*, the AOB of *Procavia capensis* is located dorsocaudal to the main olfactory bulb (MOB) as shown in sagittal sections. Throughout its dorsocaudal extent, the AOB express Gαi2 (A, B) but not Gαo (D, E). MOB glomeruli express Gαo (F), but not Gαi2 (C). Interestingly, cell layering at the AOB (G, H) is not as clear as in rodents or as in its MOB (I). The dashed area in E shows the vomeronasal afferences; although Gαo is expressed to some extent at the glomerular layer (Gl) of the AOB, the vomeronasal nerve (VN) shows no Gαo expression. EP, external plexiform layer; Gr, granule cell layer; LOT, lateral olfactory tract; M/T, mitral/tufted cell layer. Dorsal is to the top and anterior is to the right. Scale bar: 500 µm in A, D and C; 200 µm in B, E and F; 100 µm in C, F and I.

The AOB of *P. capensis* also occupies a somewhat small volume relative to its prominent MOB (Figure 3A, 3D and 3G). However, unlike other Afrotheres studied, such as tenrecs [14] and elephants [8], the MOB of *P. capensis* lacks an olfactory ventricle (Figure 3G). Gαi2 expression was confined to the VN and Gl layers of the AOB, throughout their entire extent (Figure 3A and 3B). There was no Gαi2 expression in axons arriving to MOB glomeruli via the ON (Figure 3C). As in *S. beecheyi*, Primates and Laurasiatheres, Gαo protein was absent from the VN layer and showed low expression in the parenchymal portion of the AOB (Figure 3D and 3E). However, MOB glomeruli and the ON showed Gαo expression (Figure 3F). Note that MOB layers are clearly stratified (Figure 3I), as seems to be the general condition of the mammalian MOB.

## Discussion

Within the superorder Afrotheria, the Paenungulata clade, including elephants (order Proboscidea), sea cows (order Sirenia) and hyraxes (order Hyracoidea), shows remarkable sex dimorphisms in body size/shape and cranial/dental morphology across species. Hyracoidea include grazing ancestors that dominated the African Paleogene [38], [39], and its fossil record contains multiple evidence of sexual dimorphisms in dental and mandibular structures [34]. Adult, but not juvenile, *P. capensis* show male-biased sexual dimorphisms in body weight, morphometric components and fur coverage [40], [41]. Moreover, the shape and length of the incisors are notoriously different between sexes in all species of the Procaviidae family [35].

Similarly, the squirrel-related family, Sciuridae, shows different patterns of body size dimorphism ranging from male-biased dimorphisms in ground squirrels to female-biased dimorphisms in chipmunks [36]. Indeed, amongst 14 genera from 3 families (Sciuridae, Muridae and Heteromyidae), the genus Spermophilus (ground squirrels) shows the most pronounced sexual size dimorphisms, expressed as female/male body weight ratio (0,77±0,03; mean ± SE, see [36] and Supplementary Table S1). Indeed, *S. beecheyi* often performs bipedal visual examination after social calls [42] and responds differentially to the visual presentation of conspecifics of the same or opposite sex [43]. Moreover, behavioural displays have been observed in wild *S. beecheyi* in same sex dyads only (88% males vs. 12% females), often associated to an aggressive context [37], further suggesting that visual cues alone are sufficient to signal gender in this species.

**Figure 4:**
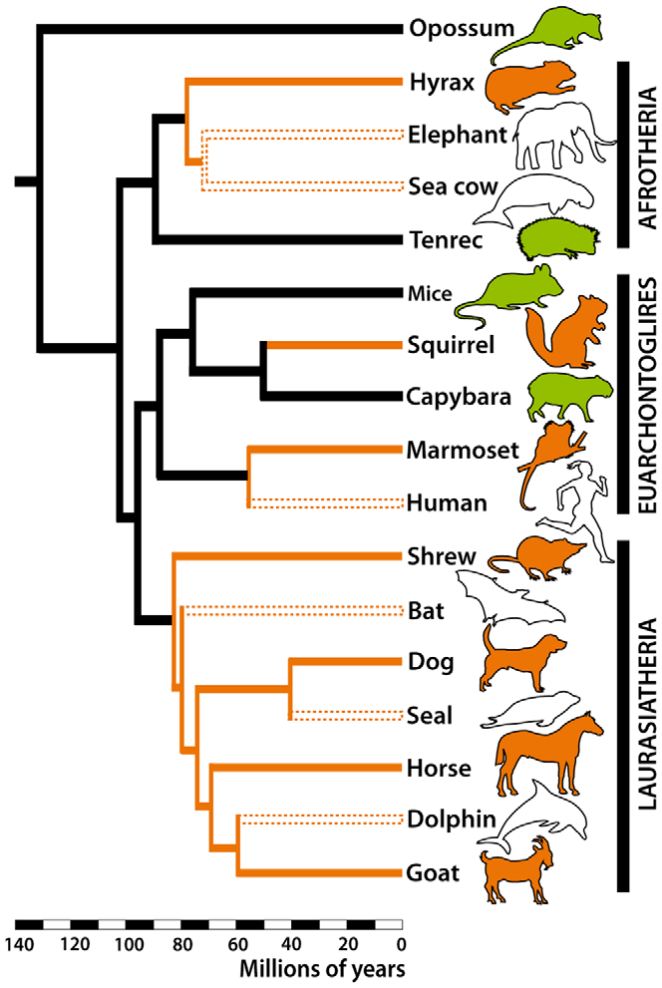
Phylogenetic tree of Eutheria showing approximate divergence times based on refs. [**44]**, [45], [46****]. At least four independent events of deterioration of the Gαo -pathway in mammals may have occurred in the lineages leading to hyraxes, squirrels, Primates and Laurasiatheres (orange lines). Note that the species that lost the complete VNS (blank silhouettes and dashed lines) are nested in lineages that first lost the Gαo-pathway. Green silhouettes represent species with both Gαi2 and Gαo vomeronasal pathways.

We have shown here that the Gαo-expressing pathway is absent in the AOB of species from two dimorphic lineages outside Primates and Laurasiatheria. Thus, considering current phylogenetic hypotheses [44], [45], [46], it can be safe to conclude that the loss of the Gαo-expressing vomeronasal pathway has occurred at least four times independently in mammals (Fig. 4). Whether these events of loss are related to the evolution of a gender recognition system based on non-vomeronasal cues (such as visual or auditory) is a possibility that deserves consideration.

Studies involving early-genes expression suggest that the Gαi-positive AOB subdomain responds predominantly in male-female interactions, while the Gαo-positive subdomain shows predominant activation in same-sex aggressive interactions, mostly between males [26], [47], [48], [49], [50], [51], [52]. Thus, we reasoned that if males could detect other males at distance, thereby reducing the risk of aggression associated to body-contact sniffing, then the Gαo-expressing pathway might undergo a relaxation of its function, which could eventually lead to its complete deterioration.

Interestingly, the onset of pseudogenisation of the vomeronasal transduction channel (TRPC2) in Old World monkeys and apes occurred by the time of acquisition of trichromatic vision [53], [54], possibly associated to the ability of assessing the reproductive status of conspecifics through the colouration and swelling of the face and genitalia, supporting a case of sensory replacement in the assessment of reproductive status.

An interesting exception of our hypothesis is observed in dimorphic capybaras. Although males are heavier than females and show a prominent gland in their snout not seen in females, the Gαo-expressing pathway is not only present but has more glomeruli than the Gαi2-expressing AOB [3]. We believe it reflects additional ecological constraints related to capybaras semiaquatic habits and/or male-male chemosignalling, plus a reduced dependence on vision associated to dense vegetation and their cathemeral/crepuscular habits. Anyhow, it is interesting to note that all species that underwent a complete loss the VNS belong to lineages that first lost the Gαo-expressing pathway (Figure 4). Interestingly, the differentiation of developing vomeronasal neurons into one of each pathway is under control of the transcription factor Ctip2/Bcl11b, and mice lacking this protein show a deficit in the Gαo-expressing pathway and an increase in the Gαi2-positive neurons [55]. Possibly, the selective loss of the Gαo-expressing pathway is related to alterations in similar regulatory mechanisms.

The origin of sexual dimorphisms has been attributed either to the mating system [31], in which monogamous species show less dimorphisms than polygynic species subjected to higher levels of male competence and/or mate choice [56], [57], [58], to different ecological niches between sexes, such as a dimorphic diet or habits [59], [60], or to differential parental care towards one sex over the other [61], [62]. However, in spite of their origin or form, we propose here that sexual dimorphisms may be phylogenetically conserved inasmuch as they allow the assessment of conspecifics' gender at distance, especially for male-male recognition. Thus, the conservation of a dimorphic system of gender recognition may allow the deterioration of the Gαo-expressing pathway by disuse. Additional comparative studies will be necessary to sustain or discard this proposition. In particular, whether additional examples of Gαo-deterioration can be found in other dimorphic lineages, such as members of Australidelphia, is an interesting possibility that deserves further investigation. These comparative studies can be further complemented with genomic enquiries on the V2R receptor family, as more genomic sequences are becoming available, especially for less accessible or endangered species.

## Materials and Methods

### Ethics Statement

All brain tissue used here was obtained from brain banks, where it was stored after being used in unrelated anatomical and physiological experiments. Thus, no ethical permits were required to undertake this study. In spite of this, all animals were treated following the protocols of the National Institute of Health Guide for the Care and Use of Laboratory animals (NIH Publications No. 80-23, 1996), and the guidelines of the University of the Witwatersrand Animal Ethics Committee. All efforts were made to minimize animal suffering.

### Tissue Processing and Immunohistochemistry

We examined olfactory bulb tissue containing the whole AOB, sent to us from laboratories that regularly perform comparative neuroanatomical studies. The olfactory bulbs of 4 adult California ground squirrels (*S. beecheyi*, two females and two males) were kindly donated to us by Dr. Felipe Fredes. Briefly, the squirrels were sacrificed with a mixture of ketamine and xylazine (200 and 12 mg/kg i.m., respectively) and perfused transcardially with 0,9% saline solution followed by 4% paraformaldehyde (PFA) in 0.1 M phosphate buffer (PB) [63]. Additionally, three adult cape hyraxes, *P. capensis*, were used in this study. Two females were euthanized with an overdose of anaesthetics (200 mg/kg sodium pentobarbital, i.p.) followed by transcardial perfusion of 0.9% saline solution and 4% PFA in PB, using the protocols, permits and ethical considerations described previously [64]. We also examined olfactory bulb tissue from a 14-month old male cape hyrax (*P. capensis*) that died in the Cleveland Zoo (kindly sent to us by Drs. Chet Sherwood and Christopher Bonar). The tissue was immersed in 10% formalin for 10 days before transferred to 0.1 M PBS with 0.1% sodium azide.

The olfactory bulbs of all specimens were transported by air to our lab in Chile for processing. Briefly, we submerged the tissue in 30% sucrose solution in PBS (w/v) until they sank (1–3 days). Then, we obtained 40 µm sagittal sections using a freezing microtome. Every other section was mounted for cresyl violet staining and the rest were used for immunohistochemistry.

We performed immunohistochemistry against Gαi2 and Gαo protein in the AOB as described previously [2], [14]. Briefly, free-floating sections were incubated in phosphate buffered saline (PBS) with 0.05% Triton X-100 (PBST) and 0.3% H_2_O_2_ at 25°C for 30 min, followed by in 3% normal goat serum (NGS) in PBST overnight. Then, they were incubated in primary immunoglobulins against Gαi2 (1∶200, mouse monoclonal, cat no. sc-13534, Santa Cruz Biotechnology, Santa Cruz, CA) or Gαo (1∶200 mouse monoclonal, cat no. sc-13532, Santa Cruz Biotechnology, Santa Cruz, CA) with 3% NGS in PBST for 3 days at 25°C. The sections were then rinsed in PBS and incubated in biotinylated goat anti-mouse secondary antibodies (1∶200, cat no. sc-2039, Santa Cruz Biotechnology, Santa Cruz, CA) for 2 hours and processed with the avidin-biotin complex (ABC Elite kit; Vector laboratories). Then, the sections were reacted in PBS with 0.6 mg/ml of 3,3-diaminobenzidine (Sigma) and 0.003% H_2_O_2_ for 20–180 sec. Sections were observed under the microscope (BX60; Olympus Optical, Thornwood, NY) and photographed with SPOT camera and software (Spot Advanced; Diagnostic instrument, Sterling Heights, MI). All figures were prepared for presentation purposes with Adobe CS3 Photoshop and Illustrator (Adobe Systems, San Jose, CA).

## Supporting Information

Abstract S1 Abstract in Spanish. Resumen en castellano.
(DOC)

Table S1 **Ratios of sexual dimorphism (female∶male) in 14 genera of Old World rodents.** Data presented as a female∶male (f∶m) ratio in body weight and/or length. Prepared with data published in ref [36].
(DOC)
