# Supplementary material for: Deterioration of the Gαo Vomeronasal Pathway in Sexually Dimorphic Mammals
Source: PLoS One. 2011 Oct 19;6(10):e26436. doi: 10.1371/journal.pone.0026436 (PMC3198400; doi:10.1371/journal.pone.0026436)
Supplement: Abstract S1 — Abstract in Spanish. Resumen en castellano. (DOC) [file pone.0026436.s001.doc]

**Resumen en castellano**

Las conductas sociales y sexuales en mamíferos están mediadas en gran medida por el sistema vomeronasal (SVN). El bulbo olfatorio accesorio (BOA) es el primer sitio de relevo sináptico del SVN y varía desde tamaños relativamente grandes en roedores Caviomorfos a pequeños en carnívoros y ungulados, hasta su total ausencia en simios, algunos murciélagos y mamíferos acuáticos.

En zarigüeyas, tenrecs, roedores y lagomorfos se han descrito dos vías vomeronasales; dos poblaciones de neuronas sensoriales expresan, ya sea la proteína Gαi2 o Gαo, envían proyecciones a la porción rostral o caudal del BOA y responden mayoritariamente ante estimulación con sustancias volátiles o no volátiles respectivamente. La vía que expresa Gαo, sin embargo, se encuentra ausente en monos titíes y en varias especies del superorden Laurasiateria. Hasta donde sabemos, no se han propuesto hipótesis que se relacionen con la pérdida de dicha vía sensorial.

Nosotros notamos que prácticamente todas las especies, tanto de Primates como de Laurasiatheria, presentan dimorfismos sexuales visualmente conspicuos. Luego propusimos que eventos similares de deterioro sensorial de la vía que expresa Gαo podrían haber ocurrido de manera convergente en otros linajes dimórficos. Estudiamos la expresión de proteínas G en el BOA de dos especies que independientemente desarrollaron dimorfismos sexuales: la ardilla terrestre de California, *Spermophilus beecheyii* (Rodentia; Sciurognathi), y el damán de las rocas, *Procavia capensis* (Afrotheria; Hyracoidea). Encontramos que ambas especies muestran una expresión homogénea de la proteína Gαi2 en todos los glomérulos del BOA, mientras que la expresión de Gαo se restringió exclusivamente a glomérulos del bulbo olfatorio principal.

Nuestros resultados sugieren que la degeneración de la vía vomeronasal que expresa Gαo ha ocurrido independientemente al menos cuatro veces en Eutheria, posiblemente relacionada al desarrollo de dimorfismos sexuales y de la habilidad de detectar el sexo de conspecíficos a distancia mediante claves no vomeronasales.
