# Supplementary material for: Deterioration of the Gαo Vomeronasal Pathway in Sexually Dimorphic Mammals
Source: PLoS One. 2011 Oct 19;6(10):e26436. doi: 10.1371/journal.pone.0026436 (PMC3198400; doi:10.1371/journal.pone.0026436)
Supplement: Table S1 — Ratios of sexual dimorphism (female∶male) in 14 genera of Old World rodents. Data presented as a female∶male (f∶m) ratio in body weight and/or length. Prepared with data published in ref [36]. (DOC) [file pone.0026436.s002.doc]

**Table S1.**

**Ratios of sexual dimorphism (female:male) in 14 genera of Old World rodents.**

| Family | Genera | Nº species analyzed | Mean f:m ratio | Standard error |
| --- | --- | --- | --- | --- |
| Sciuridae | Tamias | 21 | 1,03 | 0,01 |
|  | Sciurus | 5 | 1,05 | 0,06 |
|  | Marmota | 4 | 0,85 | 0,07 |
|  | Spermophilus | 13 | 0,77 | 0,03 |
|  | Cynomys | 4 | 0,81 | 0,05 |
| Muridae | Microtus | 13 | 0,92 | 0,02 |
|  | Clethrionomys | 5 | 1,07 | 0,04 |
|  | Akodon | 4 | 0,96 | 0,01 |
|  | Neotoma | 8 | 0,90 | 0,02 |
| Heteromyidae | Dipodomys | 20 | 0,99 | 0,03 |
|  | Perognathus | 8 | 0,98 | 0,01 |
|  | Chaetodipus | 14 | 0,98 | 0,01 |
|  | Heteromys | 7 | 0,95 | 0,01 |
|  | Liomys | 5 | 0,94 | 0,01 |

Data presented as a female:male (f:m) ratio in body weight and/or length.
